# Modelling a Compensation Standard for a Regional Forest Ecosystem: A Case Study in Yanqing District, Beijing, China

**DOI:** 10.3390/ijerph15040565

**Published:** 2018-03-21

**Authors:** Tan Li, Qingguo Zhang, Ying Zhang

**Affiliations:** 1 Department of Statistics, School of Science, Anhui Agricultural University, Hefei 230036, China; qgzhang@ahau.edu.cn; 2 School of Economics and Management, Beijing Forestry University, Beijing 100083, China; zhangylh@126.com

**Keywords:** forest ecosystem service, benefit theory, cost theory, ecological compensation

## Abstract

The assessment of forest ecosystem services can quantify the impact of these services on human life and is the main basis for formulating a standard of compensation for these services. Moreover, the calculation of the indirect value of forest ecosystem services should not be ignored, as has been the case in some previous publications. A low compensation standard and the lack of a dynamic coordination mechanism are the main problems existing in compensation implementation. Using comparison and analysis, this paper employed accounting for both the costs and benefits of various alternatives. The analytic hierarchy process (AHP) method and the Pearl growth-curve method were used to adjust the results. This research analyzed the contribution of each service value from the aspects of forest produce services, ecology services, and society services. We also conducted separate accounting for cost and benefit, made a comparison of accounting and evaluation methods, and estimated the implementation period of the compensation standard. The main conclusions of this research include the fact that any compensation standard should be determined from the points of view of both benefit and cost in a region. The results presented here allow the range between the benefit and cost compensation to be laid out more reasonably. The practical implications of this research include the proposal that regional decision-makers should consider a dynamic compensation method to meet with the local economic level by using diversified ways to raise the compensation standard, and that compensation channels should offer a mixed mode involving both the market and government.

## 1. Introduction

The indirect value of forest ecosystem services is an important consideration for appropriate compensation standards, which has become the consensus of different countries around the world. In America and many European countries, long-term research has been carried out concerning the issue of the evaluation of forest ecosystem services and their compensation. Governments and scholars in this research area have focused mainly on the strategy of payment for ecosystem services (PES) and have studied the relationship between forest ecosystem services and compensation standards. PES was first used to connect the ecosystem with beneficial social activities and to determine the estimated cost to replace lost ecosystem services [1,2]. In the first comprehensive multi-scale evaluation of the global ecosystem [3], ecosystem services were classified into four types: production services, regulation services, cultural services, and support services. With each term defined from the perspective of supply or demand, market and non-market values are recognized in both spatial and temporal dimensions. It is optimal to decide compensation by considering market value because doing so clarifies the calculation method, the evaluation result, the application to general issues, and whether or not the market would support the ecosystem service [4]. Forest ecosystem services undergo temporal and spatial changes, which require in-depth research on the multiple service features [5,6]. Research in this area has focused on three aspects: (1) the correct assessment of ecosystem services; (2) the analysis of the relationships among all services; and (3) the study of the issues using proper spatial and temporal scales. To sum up, the published research has generally centered on the theory and evaluation method of forest ecosystem services, the market value of services, and the contributing factors, with growing interest in the study of the relationship between forest ecosystem services and the compensation standard. What has been less studied in the area of forest ecosystem services are the conflicting evaluation methods, inconsistent evaluation criteria, and the absence of an explicit theoretical basis.

In China, the evaluation of forest ecosystem services started in the 1980s, with substantial work undertaken on the standardization and quantification of the evaluation theory. A comprehensive evaluation of China’s forest resources was done by focusing on the ecological significance of water conservation, biodiversity conservation, and air purification [7]. China has implemented national policies on payments for ecosystem services. A preliminary evaluation of China’s programs from the perspective of ecosystem services has been conducted [8], followed by a comprehensive review of ecosystem services and their economic valuation in China [9]. The evaluation of China’s forest ecosystem services proved that their value was well above the value of the corresponding forest and wood products [10,11]. Systematic research was conducted on the relationship between forest ecosystem services and forest eco-compensation that argued that eco-compensation was an effective transfer mechanism to internalize the externalities of forest ecosystem services and was an important component of China’s forestry development [12]. With economic approaches such as the replacement-cost method, opportunity-cost method, and shadow-engineering method, the indirect use value of the ecosystem of Beijing has been evaluated [13]. In recent years, there has been a growing interest in China’s academic circles in the relationship between these services and the compensation standard. In China, researchers have a universal viewpoint that the existing compensation standard is too low to remedy the forest farmers’ losses. Therefore, evaluation methods have been compared; the spatial transfer on ecological compensation has also been studied; and some studies have considered that the compensation standard should be based on the cost while others on the benefit [14].

In terms of research scale, the evaluation of forest ecosystem services has covered most of China’s provinces and their governing counties and cities, but the different evaluation methods that have been adopted make it difficult to compare the results in different regions. For previous literature, upon analyzing the characteristics of the overall forest ecosystem services and compensation standard, it was found that these studies evaluated the forest ecosystem services without benefit or cost, respectively. To address this problem, the State Forestry Bureau formulated the Forest Standard of the People’s Republic of China—Evaluation Standard on the Function of Forest Ecosystem Services (LY/T 1721-2008) in May 2008 to standardize the data source, indicator system, and evaluation formula for forest ecosystem services. Despite this effort, research on the relationship between the forest ecosystem services and the compensation standard remains insufficient. In terms of research method, research analysis from mathematical and statistical perspectives is not common. 

This paper aimed to assess the development results of forests in Yanqing District, Beijing, in order to monitor the changes in forest stock and to gain a correct understanding of the forest produce services, ecology services, and society services of the forests in this area. In this research, we used both cost theory and benefit theory to evaluate the forest ecosystem services in Yanqing District based on local forest data. The evaluation results, taking into account economic and social development as well as the existing compensation standard, helped to reveal the true importance of Yanqing’s forest ecosystem services to local economic and social development. The appropriate evaluation of these services is of great significance in implementing ecological compensation policy for forests and in fostering the correct awareness of eco-civilization among the public. What is the best way to establish a compensation mechanism in China? One way is to use a market mechanism based on the Coase theorem. Another way is for the government, as the leader, to determine the compensation. Based on this, this study investigated the coupling relationship between the economic value of forest ecosystem services and the current eco-compensation standard. 

The main content of our study is as follows. In Section 3, we assign appropriate methods for forest ecosystem services in Yanqing District and determine specific coefficients to evaluate changes in ecosystem services in the study area; in Section 4 and Section 5 we adjust the results of benefit and cost, calculate the compensation standard according to the study region economic situation, and predict the implementation time. Finally, in Section 6 we provide advice to support regional sustainable development policies.

## 2. Study Area and Data Sources 

This study focused on the Yanqing District of Beijing, an extensive, unique, geological area on the north-west fringe of Beijing. Yanqing District has been recognized as a “National Green County” and “National Garden County”. It is known as an ecological civilization pioneer in China. In 2013, this area was included in the United Nations Educational, Scientific and Cultural Organization (UNESCO) world geological park directory.

### 2.1. Natural Conditions

**Figure 1:**
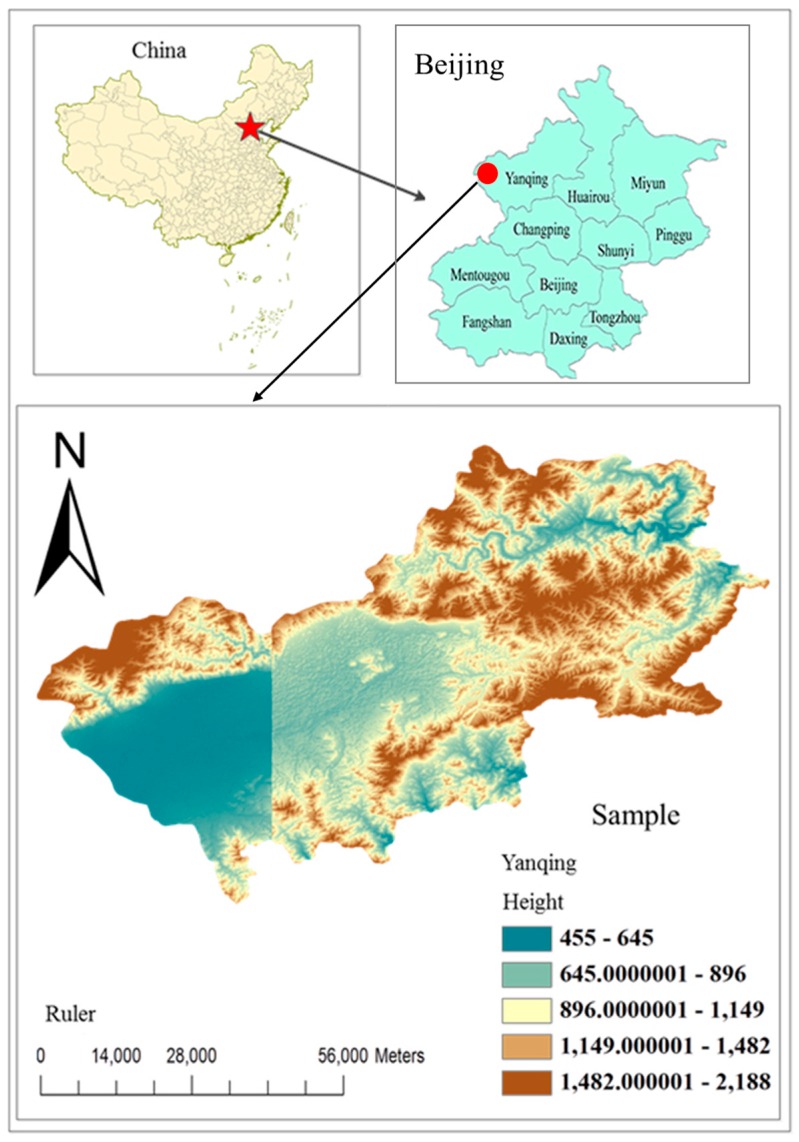
Digital elevation map of Yanqing.

The Yanqing District of Beijing (40°16′–40°47′ N, 115°44′–116°34′ E) is situated at the intersection of the Inner Mongolian Plateau and the North China Plain, 80 km from the north-west suburb of Beijing (Figure 1). It is a typical basin surrounded by mountains on three sides and by the water of the Guanting Reservoir on its south-west side.

With an average elevation of over 500 m, Yanqing covers a total area of 1993.75 km^2^, with a mountainous area accounting for 72.8%, plain area of 26.2%, and water area of 1% [15]. Located in the transitional zone between the warm temperate zone and mid-temperate zone, and between the semi-arid zone and semi-humid zone, Yanqing enjoys a continental monsoon climate. The average annual precipitation of the district reaches 467 mm. Due to the airflow from Bashang, Hebei, and the Inner Mongolian Plateau, the wind power of Yanqing is quite strong, with an annual average wind speed of 5.1 m/s. A part of the Haihe Basin, Yanqing boasts 18 rivers above Grade Four including river systems such as the Yongding River, the Chaobai River, and the North Canal.

In terms of tree composition, the district forest consists of 10 major varieties of trees (Table 1). In terms of tree structure, the district forest is classified into pure forest and mixed forest. Among the forestland area, the forest covers an area of 112,170.3 ha, 74.2% of the forestland area, while the shrubland area totals 20,720.7 ha, 13.7% of the forestland area. The maximum forest areas are: (1) protection forests (61.7%); (2) afforestation forests (shrub) (11.0%); (3) economic forests (7.0%); and (4) young forests (6.4%), totally accounting for 86.1% of the district’s forestland. The district enjoys forest coverage of 54.91% and boasts seven parks above the district level. A total of 87.5% of the forest area in the district, or 135,000 ha, are for public benefit while 10,000 ha are for commercial use. 

### 2.2. Social and Economic Situation 

#### 2.2.1. Economic Situation

In 2014, Yanqing District achieved a gross regional production of 9.98 billion Yuan (1 USD ~ 6.9 Yuan) including 968 million Yuan in the primary industry (9.7% of the total output value), 2.76 billion Yuan in the secondary industry (27.7% of the total output value), and 6.25 billion Yuan in the tertiary industry (62.6% of the total output value). In 2014, the annual fiscal revenue was 1.139 billion Yuan and the fiscal expenditure was 6.02 billion Yuan. The per capita disposable income of urban residents was 33,778 Yuan, up 8.5% from the previous year, whereas the per capita consumption expenditure totaled 19,808 Yuan, 9.1% higher than the year before [15].

In 2014, the rural economic income totaled 13.06 billion Yuan, showing an annual growth of 10.7%. The agricultural production value stood at 2.41 billion Yuan, down 7.6% on a yearly basis. The per capita net income for rural residents was 17,017 Yuan, up 9.8% from the previous year. The per capita annual living expenditure was 11,190 Yuan, recording an increase rate of 10.9%.

#### 2.2.2. Social Situation

Statistical data from local websites [15] says the population of the district totaled 316,000 at the end of 2014 including 163,000 agricultural workers (51.4%) and 153,000 non-agricultural workers (48.6%). Thanks to the wide participation of local people and vigorous support from the district government and people from all walks of life, the district’s cultural facilities have witnessed great improvement with rich cultural activities as well as enhanced protection and utilization of local cultural heritage. According to the latest statistical data, there are 430,000 books in the Yanqing district library and 133 key cultural relic sites under protection above the district level.

### 2.3. Implementation of Ecological Compensation

Starting in December 2004, an ecological compensation mechanism was implemented in Yanqing in a comprehensive way pursuant to the general plan of Beijing. At present, the ecological forests of the district under eco-compensation cover an area of 128,700 ha, involving an annual compensation fund of 44.88 million Yuan for 15 towns and 360 administrative villages. There are 8949 permanent ecological forest rangers across the district, and the highest annual salary among forest rangers reaches 7500 Yuan [15].

In terms of input of compensation funds, over 1 million Yuan in 11 towns including Zhangshanying, Jiuxian, Xiangying and Qianjiadian has been invested since 2005. Twenty-three ecological forest-management stations have been set up, having a total floor area of 790 square meters. Since the implementation of the ecological forest compensation mechanism in mountainous areas, over 20,000 farmers have been employed due to the ecological forest management and protection initiative, and the per capita monthly income of the local agricultural population has increased by over 200 Yuan.

### 2.4. Data Sources

Data for this research came from the Statistical Yearbook of Yanqing District [15], a farm-household questionnaire designed for this research, a questionnaire launched by the Forestry Administrative Department, a questionnaire for tourists, and a questionnaire for experts who study forest ecosystem services. Due to the renewal period of statistics, the data base year of 2014 was adopted for this research. Several parameters in the model are shown in Table A1 in Appendix A.

## 3. Research Methodology

This research calculated the ecosystem services value of forests in Yanqing District, considering the two aspects of benefit and cost. The ecological value Equations (1)–(8) came mostly from the People’s Republic of China Forestry Standard—Regulation for Evaluation of Ecosystem Service and Function of Forest (LY/T 1721—2008) [16,17], which was enacted by the State Forestry Administration in May 2008, although some adjustments have been made to the methods of evaluation.

In Section 3.1, we discuss the total value of forest ecosystem services including the economic benefit of forest production, ecological benefit of forest ecosystem services, and social benefit provided by this use of the forest ecosystem.

In Section 3.2, we discuss the total costs of forest ecosystem services involving direct costs, indirect costs, and opportunity costs.

### 3.1. Benefit

Benefits mainly come from economic usage which can be obtained from the statistics. Some benefits of the forestland come from ecological value (Section 3.1.1) which includes water conservation (Equation (1)), water purification (Equation (2)), soil reinforcement (Equation (3)), carbon fixation (Equation (4)), oxygen release (Equation (5)), atmosphere purification (Equation (6)), crop/pasture increases due to forest protection (Equation (7)), protecting biodiversity (Equation (8)), and forest recreation income from visitors (Equation (9)). Benefits also come through social value (Section 3.1.2), which includes income/value brought by forestland through scientific research (Equation (9)), employment (Equation (10)), health value (Equation (11)), and social development (Equation (12)).

#### 3.1.1. Determining Ecological Benefit 

(1) Water Conservation

The calculation reflects the relationship between water resource consumption and economic/social development due to systematic interactions between anthropogenic production, consumption activity, and water resources. The market value method is used in the study to evaluate the value of forest material production services. The market value method measures the economic benefits or losses of changes in environmental quality by using changes in regional output or profits caused by environmental quality changes [18]. 

The value of the annual water content regulation of a forest stand (Yuan/yr) is:

(1) $$U_{t}=10C_{k}\times A(P-E-C)$$

where *C*_k_ is unit volume income of the reservoir operation (Yuan·m^−3^); *A* is the area of the forest stand (ha); *P* is precipitation (mm/yr); *E* is evaporation of the forest stand (mm/yr); and *C* is surface runoff (mm/yr).

For the water purification value, we used the method of price substitution of tap water, which means the value is calculated using the tap water price [17]. The formula is:

(2) $$U_{s}=10K\times A(P-E-C)$$

where *K* is the price of tap water (Yuan/t); and the other indicators are as above.

(2) Soil Reinforcement 

Soil reinforcement and fertility maintenance are normally considered together [19]. Soil reinforcement means reducing soil erosion. So we compare the non-forestland and forestland to calculate the net productivity of the forest soil in the research area. However, the income of fertility maintenance is always small, so we omitted fertility maintenance. 

The value of soil reinforcement (Yuan/yr) is:

(3) $$U_{g}=A(C_{n}+C_{t})(\frac{X_{2}-X_{1}}{\rho })$$

where *C*_n_ is the output value of farmland (Yuan·m^−3^); *C*_t_ is the annual income after land development (Yuan·m^−3^); *X*_2_ is the soil erosion modulus of non-forest land (t·ha^−1^·yr^−1^); *X*_1_ is the soil erosion modulus of forest land (t·ha^−1^·yr^−1^); and *ρ* is the volume weight of soil (g·m^−3^).

(3) Carbon Fixation and Oxygen Release

We calculated this measure by summing the incomes of carbon fixation and oxygen release [20], then calculated the income of carbon fixation (Yuan/yr) as:

(4) $$U_{c}={\mathrm{AC}}_{c}(1.63R_{t}B_{n}+F_{t})$$

where *C*_c_ is the market price of carbon dioxide (Yuan·yr^−1^); *R*_t_ is the carbon content of carbon dioxide with the percentage of 27.27%; *B*_n_ is the net productivity of forest stand (t·ha^−1^·yr^−1^); and *F*_t_ is the annual carbon fixation content of the soil of the forest stand (t·ha^−1^·yr^−1^).

The volume of oxygen release can be calculated by the carbon dioxide fixed by the forest. We choose a method called the shadow price method (SPM) to evaluate the oxygen’s market value. The oxygen price was taken to be the market price of oxygen for medical use [21]. The equation for the value of oxygen release (Yuan/yr) is:

(5) $$U_{o}=1.19C_{o}AB_{n}$$

In this formula, *U*_o_ represents the annual oxygen release value of forest (Yuan·yr^−1^); *C*_o_ is the market price of oxygen in 2011 (which was 1200 Yuan·t^−1^), and the other indicators are as above.

(4) Atmosphere Purification 

This measure is based on the per unit area volume of pollutants and dust that are absorbed by forests and calculates the total pollutants and detained dusts assimilated by forests [17]. The value is calculated by the method of market value. The annual air purification value of the forest (Yuan/yr) formula is:

(6) $$U_{j}=\sum_{i=1}^{n}K_{i}\times Q_{i}\times A$$

where for each type *i* of pollutant (SO_2_, HF, NO_x_) and detained dusts, *K_i_* represents the cost of air purification (Yuan·kg^−1^); and *Q_i_* is the volume of annual absorption (kg·ha^−1^·yr^−1^).

(5) Forest Protection

It is of interest to know how forest protection is related to issues of social and ecological justice, exploring whether forest exploitation based on the top-down managerial model fosters an inequitable distribution of resources [22]. The value substitution method of disaster mitigation and yield increase is used to check the forest’s value in alleviating disaster and increasing production. The formula for the value of forest protection (Yuan/yr) is:

(7) $$U_{f}=\sum_{i=1}^{n}C_{i}\times Q_{i}\times A$$

where *n* represents the number of types of crop and pasture, and for each type, respectively, *Q_i_* is the increased production of that type of crop/pasture due to protection by the forest (including coastal protection forest) (kg·ha^−1^·yr^−1^); and *C_i_* is the income from the type (Yuan·kg^−1^).

(6) Biodiversity

The contingent value method (CVM) is usually used to evaluate the value of environments that have intangible benefits, and mainly uses a questionnaire survey to directly examine the economic behavior of respondents in a hypothetical market in order to obtain consumer willingness to pay for goods or services [20]. In this study, the benefits of biodiversity can also be evaluated in this way, that is, by asking similar questions such as “How much are you willing to pay each year to preserve the living wildlife in per unit area?” The average of the answers is used to be an annual income opportunity. It is satisfactory as the price range to calculate the corresponding income per unit area. The income value (Yuan/yr) of protecting biodiversity is:

(8) $$U_{b}=\sum_{i=1}^{n}S_{i}\times A$$

where *n* is the number of species and for each species *i*; and *S_i_* is the annual income opportunity per unit area (Yuan·ha^−1^·yr^−1^). As before, *A* is the area of forest stand.

(7) Forest Recreation

The research built a hypothetical market and obtained the WTP (willingness to pay) of the respondents [21]. The annual value of forest recreation was evaluated by the question in the designed questionnaire: “In order to enjoy the natural scenery, how much are you willing to pay for this tour?” If the respondents gave a positive answer, the questionnaire will further inquire the maximum amount to be paid, reasons for payment, and ways of payment. In this study, the data sources of forest recreation services valuation were based on the random sampling principle. Questionnaires were used in the field survey. A total of 300 questionnaires were handed out and 287 questionnaires (95.67%) were actually recovered. Among them, 277 questionnaires were valid, giving a 96.51% effective recovery rate of questionnaires.

This paper follows the above opinions and adopted *WTP* as the *CVM* (contingent value method) research and evaluation indicators.

(9) TNUV=E(WTP)×N

where *N* represents the number of yearly tourists; *E*(*WTP*) represents per capita *WTP*; and *TNUV* represents non-use value. 

*E*(*WTP*) can be expressed using the following model [23,24]:

(10) $$E(WTP)=\sum p_{i}B_{i}$$

where *p_i_* is the probability that the visitor will be willing to pay for Yanqing District and *B_i_* is the bid amount. The correlation between the *WTP* obtained in the questionnaire and individual social characteristic variables meeting the principles of economics is one of the methods for determining the validity of CVM [25]. 

#### 3.1.2. Social Benefit

According to the structure of the research, the social income was divided into the income of scientific research, employment, health, and social development. The income earned from these services was considered to be only the economic benefits; we did not consider the economic value of the services when they entered the local or national market, so we used the benefit transfer method [26].

The indicator is calculated by the total amount of scientific research funding as well as the transformation and income rate of such income. The formula is:

(11) $$U_{k}=N\times m\times n$$

Here, *U*_k_ represents the annual income of forestry scientific research (Yuan·yr^−1^); N is the total amount of scientific research funds for forestry (Yuan·yr^−1^); *m* is the transformation rate of scientific research fruits of forestry (%); and *n* represents the social income rate of fruit transformation (%).

The specific calculation method of employment is to count the newly increased amount of employment in combination with the average wage. The formula is:

(12) $$U_{e}=\Delta N\times w$$

where *U*_g_ represents the annual income of forestry employment (Yuan·yr^−1^); ΔN is the newly increased employment population of forestry workers that year (people·yr^−1^); and *w* is the annual average wage (Yuan·yr^−1^). 

The main calculation method for health income is:

(13) $$U_{h}=N\times \Delta Y\times G$$

Here, *U*_h_ represents the annual income of human health (Yuan·yr^−1^); N is the number of local residents (people·yr^−1^); ΔY is the extended age limit of human life span (a); and G is the limit of willingness to pay (Yuan·yr^−1^).

The formula for social development income is:

(14) $$U_{d}=\Delta G\times I$$

where *U*_d_ is the annual income of social development (Yuan·yr^−1^); Δ*G* is the annual average increase of GDP (Yuan·yr^−1^); and *I* is the influence coefficient of the existence of forest on GDP.

### 3.2. Cost

We considered the cost of producing and maintaining forestland as well as the cost of replacing forest services with other alternatives. Some of these costs were classified as direct costs (Section 3.2.1): the construction cost of forest (Equation (15)), forest production salary cost (Equation (16)), and operational/managerial costs (Equation (17)). Other costs were classified as risk costs (Section 3.2.2). The remaining costs evaluated in this section were classified as opportunity costs (Section 3.2.3).

#### 3.2.1. Direct Cost

The direct cost was dominated by the construction cost of forest, forestry production cost, and operational cost of the forestry department [27]. The specific calculation of the three parts is as follows.

(1) The Construction Cost of Forest

The construction cost of forest refers to the cost of afforestation including the input of soil preparation, nursery stock, fertilizing, laboring, and tending inputs for three years after afforestation. Data sources were the local forestry statistical yearbook. The formula for the construction cost of forest (Yuan·yr^−1^) is:

(15) $$C_{1}=\sum_{i}^{5}T_{i}$$

where *T*_1_–*T*_5_ refer to the input of soil preparation, nursery stock, fertilizing, laboring, and tending after afforestation (Yuan·yr^−1^), respectively.

(2) The Forestry Production Salary Cost

The forestry production cost includes the input cost of forest products (forest, logs, firewood, bamboo, and rattan) and non-forest products (animals and plants), and the input cost for herding in forests. This research adopted the method of production cost to unify the accounting of the two input costs. The equation for the total input cost of forestry production (Yuan·yr^−1^) is:

(16) $$C_{2}=\sum_{i}^{3}W_{i}\times N_{i}$$

where *W*_1_–*W*_3_ refer to the average salaries of workers of forest products, non-forest products, and herding in forests (Yuan·yr^−1^), respectively; and *N*_1_–*N*_3_ are the number of workers dealing with forest products, non-forest products, and herding in forests, respectively.

(3) The Non-Salary Operational Cost of the Forestry Department

The operational cost of the forestry department includes two types of expense: one is the administrative cost, referring to the newly increased funds at the district, township, and village levels which are used for reinforcing the management of public welfare forest; the other is the cost of forest protection, which refers to the salaries of forest rangers and the funds for management and protection. The accounting information of China’s national economy was the main data source and included the expenditure of the operational and managerial activities of various forest resources. The equation for the total operational cost of the forestry department (Yuan·yr^−1^) is:

(17) $$C_{3}=M+G$$

where *M* is the administrative cost of the forestry department (Yuan·yr^−1^); and *G* refers to the input of the forest protection cost (Yuan·yr^−1^).

Apart from the three specifically mentioned expenditures, there were actually some other inputs such as office expenses of the forestry managerial department and so on, but these accounted for only a small proportion and so were omitted in this calculation.

#### 3.2.2. Risk Cost

Risk costs mean the costs of the potential ecological and social economic security risks [27]. Based on this, this study mainly calculated the risk costs of accidents. In this case, risk costs mainly referred to natural hazards including plant diseases and forest fire disasters. This measure mainly referred to the treatment expenses of forest plant diseases as well as the management expenditure of insect pests. In addition, costs were added such as forest fire insurance and the wages of the rangers. Hence, the risk costs (Yuan·yr^−1^) can be formulated as:

(18) $$C_{q}=C_{z}+C_{a}$$

where *C*_q_ refers to the risk cost; *C*_z_ is the forest management and protection expense of plant diseases and insect pests (Yuan·yr^−1^); and *C*_a_ is the cost of forest fire insurance and wages of the rangers (Yuan·yr^−1^).

#### 3.2.3. Opportunity Cost

The opportunity cost of the forest is calculated by multiplying the total area of forest by the economic output of various products per unit land area, which is just the opportunity cost of forest eco-compensation. Therefore, the measurement of opportunity cost is calculated using the economic benefits generated directly by forest products, non-forest products, and herding and hunting, which can be calculated using the cost-accounting theory system [28]. The products considered were forest products such as lumber and firewood; non-forest products including animals like bees and plants such as fungus and medical materials; and herding and hunting products. The formula for the opportunity cost (Yuan·yr^−1^) is:

(19) $$C_{m}=\sum_{i=1}^{n}P_{i}Y_{i}$$

Here, for each product *i*, *P_i_* is the price (Yuan·kg^−1^); and *Y_i_* is the annual average amount (kg·yr^−1^). 

## 4. Results 

### 4.1. Benefit Results 

#### 4.1.1. Economic Benefit 

The forestry output value of Yanqing District in 2014 was 0.22 billion Yuan. The forestry output value for non-commercial forest can be calculated based on this value and the proportion of the non-commercial forest in Yanqing, that is, 22,202.8 × 87.5% = 0.19 billion Yuan, where the unit value is 1409.07 Yuan·yr^−1^.

The output value of non-wood products mainly depends on the local forests’ economic development. Yanqing’s forest area for economic development amounts to 230 ha including forest flowers of 50 ha, forest medicinal plants of 90 ha, forest mulberry of 100,000 ha, forest fungus of 70 ha, and forest birds of 20 ha [15]. In the process of compensation, the actual computation should depend on the specific circumstances due to the complicated forest economy, and it is unnecessary to account for all conditions. Using this calculation method, the total output value of the non-wood forest products throughout 2014 was 45.5 million Yuan (unit value: 19.78 Yuan·ha^−1^) (see Table 2). An additional 1600 farmers became employed. There was an average increase in forestland revenue of 500 Yuan for each hectare. At the same time, the forest economic industry has spawned the development of related industries. According to the statistical yearbook, the local income from grazing and hunting in 2014 was negligible and thus can be omitted. For this research, this fractional income was not incorporated into the total value, which is clearly sensible because the result of the calculation is relatively large.

#### 4.1.2. Ecological Benefit

(1) Water Conservation

Benefit from water flow regulation: the annual precipitation in Yanqing District averages 467 mm as measured, with an aggregate of approx. 230 mm in evaporation and ground runoff. Therefore, the intake of the reservoirs is roughly 2.3 Yuan·m^−3^, with the benefit of regulating water flow of approx. 745 million Yuan·yr^−1^.

Water purification: the prevailing price of running water in Beijing is 4 Yuan·m^−3^. Hence, the benefit from water purification amounts to 1.28 billion Yuan·yr^−1^. 

(2) Soil and Fertility Conservation

Benefit from soil conservation: according to the Beijing Statistical Yearbook and the relevant water conservation authority, the average bulk density of soil is 1.25 per unit. The average difference in soil erosion modulus between non-forestland and forestland reaches approx. 70. The output value of farmland is approx. 50 Yuan·ha^−1^, and 10.68 Yuan·ha^−1^ after land development. With these data, the benefit from soil conservation was concluded to be 459 million Yuan. 

Benefit from fertility conservation: the organic matter in the surface soil in the local woodland averages 3%, the content of total nitrogen averages 0.19%, total phosphorus 0.02%, and total potassium 0.08%. Assuming that C_1_, C_2_, and C_3_, the prices for diammonium phosphate, potassium chloride, and organic matter are 2400 Yuan·t^−1^, 2200 Yuan·t^−1^, and 320 Yuan·t^−1^, respectively. Assuming that R_1_, R_2_, and R_3_, the nitrogen content of diammonium phosphate, the phosphorus content of diammonium phosphate, and the potassium content of potassium chloride are 14%, 15.01%, and 50%, respectively. Based on these data, we can infer the benefit from fertility conservation was approx. 462 million Yuan. 

(3) Carbon Sequestration and Oxygen Release

Benefit from carbon sequestration: With a carbon price of 1200 Yuan·t^−1^, the average value for *B*_y_ of 8.977 t·ha^−1^·yr^−1^, and *F*_s carbon_ being 0.4 t·ha^−1^·yr^−1^, we calculated that the annual profit of carbon sequestration was 711 million Yuan.

Benefit from oxygen release: the amount of released oxygen was inferred from the carbon dioxide sequestration in forests and the oxygen price for medical use was adopted. *C*_o_, the market price of oxygen in 2011, was 1200 Yuan·t^−1^. Based on these data, the benefit from oxygen release was concluded to be approx. 1.731 billion Yuan. 

(4) Air Purification

According to related statistical yearbooks, it can be inferred that the benefit from air purification by non-commercial forests in Yanqing District amounts to approx. 15 million Yuan (Table 3).

(5) Forest Protection 

According to related statistical yearbooks, it can be concluded that the annual benefit from forest protection amounts to approx. 675 million Yuan. 

(6) Biodiversity

Based on the operational income from biodiversity protection and relevant biodiversity indexes, we obtained the annual income of approx. 1.35 billion Yuan from biodiversity protection. 

(7) Forest Recreation 

According to relevant surveys on local tourist attractions and ticket proceeds, the annual income from recreation in the non-commercial forests amounts to approx. 135 million Yuan. 

#### 4.1.3. Social Benefit

The benefit from scientific research, represented by the effective benefit from scientific achievements, can be calculated from the total scientific research funds allocated for the forestland of Yanqing, the conversion rate of research achievements, and the yield rate. According to the Statistical Yearbook of Beijing and local statistical data, relevant data on Yanqing’s total scientific research funds in the forestry and conversion rate were obtained. Based on these data, we calculated Yanqing’s benefit from scientific achievements in 2014, which amounted to approx. 550,000 Yuan, a unit area benefit of 4.07 Yuan·ha^−1^.

In terms of employment benefit, Yanqing’s forestry industry employed 20,000 in 2014 according to the Yanqing Statistical Yearbook, an increase of 420 from the previous year, with an average annual salary of 4800 Yuan per person. Therefore, Yanqing’s employment benefit in 2014 was approx. 2 million Yuan, a unit area benefit of 14.8 Yuan·ha^−1^. 

In terms of health benefit, based on the local population, the average growth of life span, and the maximum payment from the local residents, we obtained the health benefit in 2014, which amounted to 7.925 million Yuan, a unit area annual benefit of 58.7 Yuan·ha^−1^. 

In terms of social development benefit, based on the average growth of GDP (12.1%) in 2014 and the coefficient of forest’s contribution to GDP, the social development benefit was approx. 121,000 Yuan, a unit area annual benefit of 0.9 Yuan·ha^−1^.

Using the above calculation, we obtained the total social benefit in Yanqing District in 2014 (Table 4). It can be seen from this table that the health benefit topped the benefit list, followed by the employment benefit, then scientific research benefit, and social development benefit.

### 4.2. Cost Results

#### 4.2.1. Direct Cost

Direct cost accounting includes the forest’s construction cost, production cost, and the operation cost. In this part, these three costs will be studied. 

(1) Construction Cost of Forest 

According to Yanqing’s Statistical Yearbook (2014) [15] and the field research data, Yanqing’s forest construction cost in 2014 totaled approx. 201 million Yuan, a unit area construction cost of 1492.54 Yuan·ha^−1^ (Table 5). 

(2) Accounting Method for Forestry Production Cost

The forestry production cost covers the cost of forest products (timber, logs, fuel wood, and bamboo rattans), non-wood forest products (animals and plants), and grazing in the forest. According to field research data and the data provided by Yanqing’s government, the production cost of Yanqing’s forestry in 2014 was 1.01 billion Yuan, a unit area production cost of 7462.69 Yuan·ha^−1^ (Table 5).

(3) Accounting Method for the Operation Cost of Forestry

According to the field research data, Yanqing’s input in this aspect in 2014 could be roughly estimated as 0.6 billion Yuan, a unit area operation cost of 4477.61 Yuan·ha^−1^ (Table 5). 

In addition to the above three specific costs, there were some other costs to be included in the direct cost, such as the office expenses of the forestry administrative departments. However, owing to their small weights when compared to the total cost, they were omitted from consideration. 

#### 4.2.2. Risk Cost

This part includes the treatment expenses of forest plant diseases as well as the management expenditure of insect pests and costs on forest fire insurance. In 2014, to sum up, the risk costs for forests primarily included two parts, as shown in Table 6.

#### 4.2.3. Opportunity Cost

In 2014, the output value of Yanqing’s forestry stood at 222 million Yuan. In light of the diverse functions of the forest ecosystem, we calculated the average loss and obtained the opportunity cost of 2.4 billion Yuan in 2014 (Table 7), a unit area opportunity cost of 1423.85 Yuan·ha^−1^. 

## 5. Compensation Standard Estimation and Discussion

### 5.1. Benefit Adjustment

According to the above calculations, the produce, ecology, and society services from the ecosystem of local forests are enormous, a unit area benefit of approx. 57,402.32 Yuan·ha^−1^, far more than the current standard compensation price (2248.88 Yuan·ha^−1^). To minimize the errors in calculations, an analytic hierarchy process (AHP) can be applied to calculate the weights of economic, social, and ecological benefits, and then these weights can be used to adjust the proportion of these benefits [29]. 

First, we used the expert assessment assignment method to analyze the weights of the three services which are the produce service, the ecology service and the society service. In the process of evaluation, we assumed the evaluation of Yanqing’s non-commercial forest benefit as objective level A. Then, we assumed the three benefits as B_1_, B_2_, and B_3_ under index level B. The economic benefit in level B was subdivided into benefit from wood products, non-wood products, and grazing and hunting. The ecological benefit was subdivided into water conservation, soil and fertility conservation, carbon sequestration and oxygen release, air purification, forest protection, biodiversity protection, and forest recreation. The social benefit was subdivided into benefits from scientific research, employment benefit, health benefit, and benefit for social development. Therefore, we used a total of 14 detailed indexes from C_1_ to C_14_ under level C (Table 8). In this research step, we invited 15 experts from the local forestry department to grade each index according to its importance. The detailed processes and results are as follows:

First, construct the judgment matrix A–B, B_1_–C_1_, B_2_–C_2_, B_3_–C_3_.

Second, determine the latent root and the eigenvector; and do a consistency check for the CR (consistency ratio) coefficient. If the result is smaller than 0.1, the consistency check is deemed to have passed. 

Third, carry out general level ranking (see Table 5 for details). It can be seen from the evaluation of Yanqing’s non-commercial forests that the weight of economic benefit accounts for 63.7%, the ecological benefit is 25.83%, and the social benefit 1 is 0.47%. Accordingly, the total unit area benefit of 57,402.32 Yuan·ha^−1^ as calculated should be adjusted to 14,473.83 Yuan·ha^−1^, which can be used as a reasonable upper limit for the ecological compensation standard for Yanqing’s non-commercial forests. This value can be used with the result obtained from the cost-based evaluation below to arrive at a reasonable compensation range.

Using the above strategies for evaluating forest ecosystem services, the benefits of non-commercial forests in Yanqing District in 2014 were calculated in a comprehensive and systematic way (Table 9). As of 2014, the gross benefit from the forest ecosystem was 78.01 billion Yuan, including 2.4 billion Yuan in forest products, 75.53 billion Yuan in services value, and 80 million Yuan in social development benefit. 

### 5.2. Cost Adjustment

During cost calculation, mistaken calculation results may appear due to the long time span between data updates. For this reason, an adjustment should be made based on a sigmoid growth curve.

(20) $$y=\frac{L}{1+ae^{-bt}}$$

In the process of calculation, assuming *L* = *a* = *b* = 1, the simplified form for the Pearl growth curve is obtained [30], namely:

(21) $$y=\frac{1}{1+e^{-t}}$$

Then, we replaced the time coordinate with the reciprocal of the local Engel coefficient of 2014 (*En*) and carried out the corresponding conversion to determine the relationship between *En* and *y*. Based on the statistical data of the local living expenditures and the local food expenditures as recorded in the Yanqing Statistical Yearbook (2014), the value of *y* was obtained, which was put into the formula to produce the adjusted value. Since the Pearl growth curve and the Engel coefficient were only relevant to the residents’ consumption regarding food expenditure, but not to other aspects such as healthcare, medical treatment, education, etc. provided by the ecosystem services, the calculation result by the Pearl growth curve was relatively large. In practice, therefore, we needed to pay attention to the expenditures incurred for health care, medical treatment, and education. 

According to the Yanqing Statistical Yearbook (2014) and Beijing Regional Statistical Yearbook (2014), Yanqing’s per capita annual living expenditure in 2014 was 8135 Yuan, the per capita expenditure on food 2859 Yuan, and the per capita expenditure on health care, medical treatment and education approx. 800 Yuan. Based on these data, the revised Engel coefficient in Yanqing was calculated to be approx. 0.45, and the value of t (–0.78) was obtained. According to the growth curve function, the coefficient y during growth period was obtained, which was 0.31. Finally, we arrived at the gross value of per unit area cost of 4949.67 Yuan·ha^−1^ in Yanqing District for the year 2014. 

Using the above strategies for evaluating forest ecosystem services, the cost of non-commercial forests in Yanqing District in 2014 was calculated in a comprehensive and systematic way (Table 10). As of 2014, the total cost was 2.06 billion Yuan including the direct cost of 18.12 billion Yuan, risk cost of 1.11 billion Yuan, and opportunity cost of 2.4 billion Yuan.

### 5.3. Compensation Standard Estimation

After adjusting the per unit area benefit and per unit area cost in evaluation practice, they became 27,753.52 Yuan·ha^−1^ and 6097 Yuan·ha^−1^, respectively, which were both well above the current compensation standards in Yanqing District. According to the local statistical yearbook in Yanqing District, the district achieved a regional GDP of 9.98 billion Yuan in 2014, with a total income of 7.80 billion Yuan from the forest ecosystem. That forest income was 78% of the GDP. Given the huge value of the forest ecosystem services, a proper evaluation of these services is called for to better accommodate the development of forestry. However, the current financial investment in Yanqing’s forestry is insufficient to meet the compensation standard, as shown by the results of this case study.

Accordingly, we assumed that a reasonable compensation standard could be set up between the cost and benefit as calculated. We extrapolated from 493 million Yuan in local financial investment in forestry for the year of 2014, 11.2% of annual average GDP growth rate in past five years, 6.5% growth rate in the forestry investment, and 1.35 billion ha of non-commercial forests. 

Therefore, we established a hypothesis: the financial investment’s growth rate was assumed as 11.2%, *Y*(*n*) equals the assumed compensation standard (Table 11), *n* equals the year after 2014, *n* = 1 (2015), 2 (2016), etc. The compensation standard range was also assumed as a 6.5% growth rate, *C*(*n*) equals the adjusted cost value (Table 10); *B*(*n*) equals the adjusted benefit value (Table 9).

(22) $$Y(n)=2248.88\;\times \;111.2{\%}^{n}$$

(23) $$C(n)=6097\times 106.5{\%}^{n}$$

(24) $$B(n)=14473.83\times 106.5{\%}^{n}$$

Having done this, and using the range of compensation standard as calculated below, it was concluded that it would take approx. 26 years (Table 11) before the government investment could exceed the cost as calculated in Table 11 to meet the compensation standard range as calculated above. Furthermore, we estimated that it would take 44 years to meet the benefit value. As the period was extended so long, we have not listed all of the tendencies for the benefit changes. These results showed that the compensation standard now is far from enough. Evaluating the long-term effects is currently not attainable; therefore, we chose this approach to simulate the tendency of forest eco-compensation. The results of such approaches will exaggerate the value of regional forest ecosystems as well as the economic significance of related measures to maintain and restore the forest ecosystem absolutely, and will cause the disruption of land-use planning, thus affecting the scientific and feasible planning of regional economic development. Compared with previous studies [21,31], the importance of forest ecosystem services in Yanqing to human welfare has been underestimated by the socio-economic system. Thus, policies on the eco-compensation of forest ecosystem services should be established to maintain the sustainable supply of the forest ecosystem services in Yanqing District, Beijing.

## 6. Conclusions

This work accounted and analyzed the benefit and the cost of forest ecosystems in order to meet compensation standards. The benefits of forest ecosystems include the economic benefit, the ecological benefit, and the social benefit, while the costs of forest ecosystems include the direct cost, risk cost, and opportunity cost. Next, the total of benefit and cost results were adjusted by AHP and the Pearl growth-curve method to obtain a closer to actual condition. With the GDP growth rate and the forestry investment growth rate, we respectively calculated that it would take 26 years for the current compensation standard to meet the adjusted cost result, and 44 years to meet the adjusted benefit result. It is of great importance to raise the compensation standard in the region and intensify forestry investment. Concrete ways to collect funds in the compensation of forest ecosystems are to receive funds from national finance, impose ecological safety insurance, etc.

It is worth noting that this Yanqing case was merely used to provide evidence for the research idea of this paper. In the actual work of scientific research, it is not necessary for all study fields to conform to every indicator used in this particular framework, calculating each one individually, because of the differences in the geographic, economic, and social conditions in other areas, and features of the woodland. Therefore, it is imperative to determine an appropriate screening of indicators in the calculation framework to take account of specific conditions in any study area, as the literature shows [32,33,34,35].

In this research, it is common to observe the phenomena that the total benefit is greater than the total cost. In actual field investigation, the input of some forest regions is greater than its output while in other forest regions the output is greater than its input. The compensation standard is reasonable as long as it is within the rational range. From previous studies, we learned that there are some deficiencies in present ecological compensation so it is worth considering how to effectively incorporate market means and formulate a more reasonable compensation standard [36,37,38,39,40]. The rational use of ecological compensation is a significant policy mechanism for developing forestry and protecting environmental sustainability not only in China, but also in other developing countries which are confronted with acute needs of both economic development and environmental protection. Therefore, the methods used in this study and the application of the results from a policy-making perspective have implications for many developing countries. This regional-scale assessment provides a basis for ecological compensation policy formulation in larger geographical areas. The indirect value of forest ecosystem services in the study area, such as water conservation, soil conservation and carbon sequestration, were greatly underestimated. Applying the techniques of this paper to other areas will reveal if such underestimation is true of many other areas. The method of the current research also indicates that we should fully promote forest protection and management and improve environmental quality by using taking into consideration the Engel coefficient in such analyses. 

To raise the compensation standard, this being the case, the local government, communities, and business entities should be mobilized to set up funds, undertake public fund-raising, and encourage private deals with the goal of improving financial investment in local forestry, minimizing the compensation gap, and maximizing the compensation for forest workers’ losses. 

Although this paper has integrated regional statistics and survey data, it is still a preliminary study due to some limitations. For example, China is a large multi-regional country where each region has special lifestyle and ecosystem characteristics, so our future work will be to examine the regional eco-compensation standard in more detail.

## Figures and Tables

**Table 1:** Statistics of the areas and volume of dominant plant species in Yanqing.

Dominant	Area/ha	Percent	Volume/m^3^	Volume Percent	Per Unit Volume m^3^/ha
pine	12,530.7	12.3	231,533.1	13.2	18.5
platycladus	7817.3	7.7	28,008.5	1.6	3.6
larch	2178.1	2.1	49,647.2	2.8	22.8
aspen	3051.9	3.0	62,399.3	3.6	20.4
oak	34,651.1	34.1	545,622.9	31.1	15.7
locust	2402.2	2.4	44,469.9	2.5	18.5
poplar	5004.6	4.9	509,265.1	29.0	101.8
birch	4610.1	4.5	131,502.5	7.5	28.5
broad-leaf	10,580.2	10.4	154,321.2	8.7	14.6
Total	101,655.8	100	1,756,769.7	100	17.3

**Table 2:** Economic income of public welfare forest in Yanqing District in 2014.

Economic Benefit	Benefit (10^8^ Yuan)	Per Area (Yuan·ha^−1^)
Forest products	1.94	1409.07
Non-forest products	0.46	19.78
Total	2.40	1423.85

**Table 3:** Ecological income in Yanqing District in 2014.

Ecosystem Services	Benefit (10^9^ Yuan)	Benefit per Area (10^4^ Yuan·ha^−1^)	Rank
Water conservation	20.25	1.5	2
Soil fertilizing	9.11	0.67	4
Carbon fixation	24.42	1.81	1
Gas purification	0.15	0.01	7
Forest protection	6.75	0.5	5
Biodiversity	13.5	1	3
Tourism	1.35	0.1	6
Total	75.53	5.59	

**Table 4:** Social benefit in Yanqing District in 2014.

Social Benefit	Benefit (10^5^ Yuan)	Benefit per Unit Area (Yuan·ha^−1^)	Rank
Science research	55	4.07	3
Employment	200	14.80	2
Health	792.5	58.70	1
Development	12.1	0.90	4
Total	804.6	78.47	

**Table 5:** Direct cost of public welfare forest in Yanqing District in 2014.

Direct Cost	Cost (10^9^ Yuan)	Cost per Unit Area (Yuan·ha^−1^)
Construction	2.01	1492.54
Production	10.07	7462.69
Operating	6.04	4477.61
Total	18.12	13,432.84

**Table 6:** Risk cost of forest in Yanqing District in 2014.

Indirect Cost	Cost (10^9^ Yuan)	Cost per Unit Area (Yuan·ha^−1^)
Plant and insect pests	2.09	1537.96
Fire insurance cost	4.11	3273.10
Total	6.21	4811.06

**Table 7:** Opportunity cost of public welfare forest in Yanqing District in 2014.

Opportunity Cost	Result (10^9^ Yuan)	Per Area (Yuan·ha^−1^)
Forest products	1.94	1409.07
Non-forest products	0.46	19.78
Total	2.40	1423.85

**Table 8:** The analytic hierarchy process (AHP) method used in the income analysis of forest in Yanqing District.

Level	B_1_	B_2_	B_3_	Rank	Relative Importance of Factor
C_1_	0.75			0.4777	
C_2_	0.25			0.1592	0.637 (weight of forest economic benefits)
C_3_		0.0953		0.0246	
C_4_		0.1404		0.0363	
C_5_		0.1404		0.0363	0.2583 (weight of forest ecological benefits)
C_6_		0.1404		0.0363	
C_7_		0.1921		0.0496	
C_8_		0.1510		0.0390	
C_9_		0.1404		0.0363	
C_10_			0.5205	0.0545	
C_11_			0.2010	0.0210	0.1047 (weight of forest social benefits)
C_12_			0.0776	0.0081	
C_13_			0.2010	0.0210	

**Table 9:** Total benefit of forest in Yanqing District in 2014.

Index	Result (10^9^ Yuan·ha^−1^)	Per Area (Yuan·ha^−1^)	Adjusted Result in per Area (Yuan·ha^−1^)
Economic benefit	0.24	1423.85	460.90
Ecological benefit	75.53	55,900	27,279.20
Social benefit	0.08	78.47	14.85
Total benefit	78.01	57,402.32	14,473.83

**Table 10:** Total cost of forest in Yanqing District in 2014.

Index	Result (10^9^ Yuan·ha^−1^)	Per Area (Yuan·ha^−1^)	Adjusted Result in per Area (Yuan·ha^−1^)
Direct cost	18.12	13,432.84	4164.18
Risk cost	6.21	4811.06	1491.43
Opportunity cost	2.4	1423.85	441.39
Total cost	26.73	19,667.75	6097

**Table 11:** Compensation standard estimation.

Year	Y (Yuan·ha^−1^)	Cost (Yuan·ha^−1^)	Year	Y (Yuan·ha^−1^)	Cost (Yuan·ha^−1^)
2014	2249	6097	2028	9941	15,623
2015	2500	6493	2029	11,054	16,709
2016	2780	6974	2030	12,292	17,870
2017	3092	7459	2031	13,669	19,112
2018	3438	7978	2032	15,200	20,441
2019	3823	8532	2033	16,902	21,862
2020	4252	9125	2034	18,795	23,382
2021	4728	9760	2035	20,901	24,901.83
2022	5257	10,438	2036	23,242	26,520
2023	5846	11,164	2037	25,845	28,244
2024	6501	11,940	2038	28,739	30,080
2025	7229	12,770	2039	31,958	32,035
2026	8039	13,658	2040	35,537	34,117
2027	8939	14,607			

## Appendix A

**Table A1:** Price parameters of Forest ecosystem service value.

Parameter Name	Information Source	Unit	Value
Investment in volume of water reservoir	average cost per unit water reservoir volume was 2.17 Yuan·t^−1^ according to the 1993–1999 yearbook of China’s water conservation and price index in 2014 was 3.3, so cost for constructing unit volume of water reservoir was 7.16 Yuan·t^−1^	(Yuan·t^−1^)	7.16 (2013)
Cost for purifying water	Replacement cost method	(Yuan·t^−1^)	1.05 (2013)
Cost for digging earthwork per unit area	Cost for the local artificial excavation	(Yuan·m^−3^)	12.6
Price for C fixation	Tax rate of Sweden for C was 1200 Yuan·t^−1^	(Yuan·t^−1^)	1200
Price for O_2_ production	Industrial oxygen generation method	(Yuan·t^−1^)	1000
N content of diammonium phosphate (DAP)	Manual for chemical products	(%)	14.0
P content of DAP	Manual for chemical products	(%)	15.01
K content of KCl	Manual for chemical products	(%)	50.0
Price of DAP	Average price during the spring of 2014	(Yuan·t^−1^)	2400
Price of KCl	Average price during the spring of 2014	(Yuan·t^−1^)	2200
Price of organic matter	Average price during the spring of 2014	(Yuan·t^−1^)	320
Price for SO_2_ treatment	Market price method	(Yuan·kg^−1^)	1.2
Price for fluorochemical treatment	Market price method	(Yuan·kg^−1^)	0.69
Price for NO_x_ treatment	Market price method	(Yuan·kg^−1^)	0.63
Price for cleaning dust	Market price method	(Yuan·kg^−1^)	0.15

## References

[1] Costanza R., dArge R., deGroot R., Farber S., Grasso M., Hannon B., Limburg K., Naeem S., ONeill R.V., Paruelo J. (1997). The value of the world’s ecosystem services and natural capital. Nature.

[2] Ghaley B.B., Vesterdal L., Porter J.R. (2014). Quantification and valuation of ecosystem services in diverse production systems for informed decision-making. Environ. Sci. Policy.

[3] Assesment M.E. (2005). Ecosystems and Human well-being: Biodiversity synthesis. World Resour. Inst..

[4] Daily G.C., Polasky S., Goldstein J., Kareiva P.M., Mooney H.A., Pejchar L., Ricketts T.H., Salzman J., Shallenberger R. (2009). Ecosystem services in decision making: Time to deliver. Front. Ecol. Environ..

[5] Patterson T.M., Coelho D.L. (2009). Ecosystem services: Foundations, opportunities, and challenges for the forest products sector. For. Ecol. Manag..

[6] Engel S., Pagiola S., Wunder S. (2008). Designing payments for environmental services in theory and practice: An overview of the issues. Ecol. Econ..

[7] Niu X., Wang B., Liu S., Liu C., Wei W., Kauppi P.E. (2012). Economical assessment of forest ecosystem services in China: Characteristics and implications. Ecol. Complex..

[8] Liu J.G., Li S.X., Ouyang Z.Y., Tam C., Chen X.D. (2008). Ecological and socioeconomic effects of China’s policies for ecosystem services. Proc. Natl. Acad. Sci. USA.

[9] Zhang B.A., Li W.H., Xie G.D. (2010). Ecosystem services research in China: Progress and perspective. Ecol. Econ..

[10] Liu J., Ouyang Z., Pimm S.L., Raven P.H., Wang X., Miao H., Han N. (2003). Protecting China’s Biodiversity. Science.

[11] Wang D., Wang B., Niu X. (2014). Forest carbon sequestration in China and its benefits. Scand. J. For. Res..

[12] Liu Y., Li J., Zhang H. (2012). An ecosystem service valuation of land use change in Taiyuan City, China. Ecol. Model..

[13] Zhang B.A., Xie G.D., Zhang C.Q., Zhang J. (2012). The economic benefits of rainwater-runoff reduction by urban green spaces: A case study in Beijing, China. J. Environ. Manag..

[14] Ouyang Z., Zheng H., Xiao Y., Polasky S., Liu J., Xu W., Wang Q., Zhang L., Xiao Y., Rao E. (2016). Improvements in ecosystem services from investments in natural capital. Science.

[15] Yanqing County Bureau of Statistics (2014). Statistical Yearbook of Yanqing County.

[16] Guo H., Wang B., Ma X.Q., Zhao G.D., Li S.N. (2008). Evaluation of ecosystem services of Chinese pine forests in China. Sci. China Ser. C.

[17] National Forestry Bureau of China (2008). Specifications for Assessment of Forest Ecosystem Services in China.

[18] Ma D., Xian C., Zhang J., Zhang R., Ouyang Z. (2015). The Evaluation of Water Footprints and Sustainable Water Utilization in Beijing. Sustainability.

[19] Robinson D.A., Fraser I., Dominati E.J., Davíðsdóttir B., Jónsson J.O.G., Jones L., Jones S.B., Tuller M., Lebron I., Bristow K.L. (2014). On the Value of Soil Resources in the Context of Natural Capital and Ecosystem Service Delivery. Soil Sci. Soc. Am. J..

[20] Zhang Y., Chen J., Hu M., Offer A. (2016). Valuation of forest carbon sinks in China within the framework of the system of national accounts. J. For. Res..

[21] Xie G.D., Li W.H., Xiao Y., Zhang B.A., Lu C.X., An K., Wang J.X., Xu K., Wang J.Z. (2010). Forest Ecosystem Services and Their Values in Beijing. Chin. Geogr. Sci..

[22] Kopnina H. (2016). Commodification of natural resources and forest ecosystem services: Examining implications for forest protection. Environ. Conserv..

[23] Hanemann W.M. (1989). Welfare Evaluations in Contingent Valuation—Experiments with Discrete Response Data: Reply. Am. J. Agric. Econ..

[24] Ao C., Li Y., Feng L., Jiao Y. (2010). Evaluating the non-use value of Sanjiang wetland based on contigent valuation method. Acta Ecol. Sin..

[25] Park S., Martin A. (2007). A novel assessment tool for reusability of wastes. J. Hazard. Mater..

[26] Uddin M.S., Steveninck E.D.R.V., Stuip M., Shah M.A.R. (2013). Economic valuation of provisioning and cultural services of a protected mangrove ecosystem: A case study on Sundarbans Reserve Forest, Bangladesh. Ecosyst. Serv..

[27] Cao S., Li Y., Lu C. (2016). A measure of the net value of ecosystem services and the evaluation of Beijing Plain Afforestation Project. Chin. Sci. Bull..

[28] Zhang J., Zhao T., Jiang C., Cao S. (2016). Opportunity cost of water allocation to afforestation rather than conservation of natural vegetation in China. Land Use Policy.

[29] Li C., Xu G., Li B., Cai F., Guo B., Tian P., Sun G., Ban Z. (1996). Decision-making Analysis of AHP for Grassland Ecosystem in Low Damp Land of Sanjiang Plain. Syst. Sci. Compr. Stud. Agric..

[30] Winsor C.P. (1932). The Gompertz curve as a growth curve. Proc. Natl. Acad. Sci. USA.

[31] Deng H., Zheng P., Liu T., Liu X. (2011). Forest ecosystem services and eco-compensation mechanisms in China. Environ. Manag..

[32] Dobbs C., Kendal D., Nitschke C.R. (2014). Multiple ecosystem services and disservices of the urban forest establishing their connections with landscape structure and sociodemographics. Ecol. Indic..

[33] Ninan K.N., Inoue M. (2013). Valuing forest ecosystem services: What we know and what we don’t. Ecol. Econ..

[34] Vo Q.T., Kuenzer C., Vo Q.M., Moder F., Oppelt N. (2012). Review of valuation methods for mangrove ecosystem services. Ecol. Indic..

[35] Ojea E., Martin-Ortega J. (2015). Understanding the economic value of water ecosystem services from tropical forests: A systematic review for South and Central America. J. For. Econ..

[36] Gómez-Baggethun E., Groot R.D., Lomas P.L., Montes C. (2010). The history of ecosystem services in economic theory and practice: From early notions to markets and payment schemes. Ecol. Econ..

[37] Milder J.C., Scherr S.J., Bracer C. (2010). Trends and future potential of payment for ecosystem services to alleviate rural poverty in developing countries. Ecol. Soc..

[38] Seidl R., Spies T.A., Peterson D.L., Stephens S.L., Hicke J.A. (2016). Searching for resilience: Addressing the impacts of changing disturbance regimes on forest ecosystem services. J. Appl. Ecol..

[39] Song M., Cen L., Zheng Z., Fisher R., Liang X., Wang Y., Huisingh D. (2017). How would big data support societal development and environmental sustainability? Insights and practices. J. Clean. Prod..

[40] Zhang B.A., Li W.H., Xie G.D., Xiao Y. (2010). Water conservation of forest ecosystem in Beijing and its value. Ecol. Econ..

